# Epidemiological Study of Hospital Admissions for Food-Induced Anaphylaxis Using the Japanese Diagnosis Procedure Combination Database

**DOI:** 10.2188/jea.JE20200309

**Published:** 2022-04-05

**Authors:** Keiji Muramatsu, Hanaka Imamura, Kei Tokutsu, Kenji Fujimoto, Kiyohide Fushimi, Shinya Matsuda

**Affiliations:** 1 Department of Preventive Medicine and Community Health, School of Medicine, University of Occupational and Environmental Health, Fukuoka, Japan; 2 Department of Health Policy and Informatics, Tokyo Medical and Dental University Graduate School of Medical and Dental Sciences, Tokyo, Japan

**Keywords:** anaphylaxis, child, food hypersensitivity, shock

## Abstract

**Background:**

Food allergies are common among children, and food-induced anaphylaxis (FIA) is a serious disease with a risk of death; however, there is yet to be a large-scale epidemiological study on causative foods in Japan. The purpose of this study was to identify foods that cause FIA in Japan.

**Methods:**

We identified 9,079 patients from the Japanese Diagnosis Procedure Combination Database who were admitted for treatment for FIA from April 1, 2014 through March 31, 2017. We extracted data on patient sex, age, use of epinephrine injections on the first day, prescription for epinephrine self-injection on the day of discharge, length of stay, readmission, and causative foods.

**Results:**

The most common causative food was eggs, followed by wheat, milk, peanuts, and buckwheat. The most common causative food in each age group was eggs among 0–3-year-olds, milk among 4–6-year-olds, peanuts among 7–19-year-olds, and wheat among those aged 20 years and older. Epinephrine was used at admission among about 40%, 50%, and over 60% of cases in which the causative food was eggs; wheat, milk and peanuts; and buckwheat, respectively. The proportion of cases with a prescription for epinephrine self-injection at discharge was highest among those in which the causative food was wheat, followed by peanuts, buckwheat, milk, and eggs.

**Conclusions:**

FIA due to peanuts has become as common in Japan as it is in the West. These results suggest the importance of taking measures to prevent peanut allergies because children cannot make adequate decisions regarding food.

## INTRODUCTION

Food allergy is a common disease in children. A previous study reported that about 8% of children in the United States, Canada, the United Kingdom, and Australia have food allergies.^1^ Additionally, about 5% of children under the age of 5 years in East Asian countries and regions, such as Hong Kong, South Korea, and Taiwan, have food allergies.^2^ In Japan, an estimated 5% to 10% of infants have food allergies.^3^ Further, the prevalence of food allergies is on the rise, with medical costs associated with food allergies having increased in the past decade in Australia, Japan, China, Korea, the United States, and Norway.^2^ Evidence also suggests that there is an increase in food allergies in developing countries in Africa and Asia due to lifestyle changes and genetic factors.^4^ These results indicate the importance of prevention and the need for proper treatments for food allergies.

Anaphylactic shock is a serious allergic reaction, and food is an important causative factor. A study in Europe demonstrated that food is the most common cause of anaphylactic shock.^5^ In the United States, 40% of children with a food allergy experience severe symptoms.^6^ Food-induced anaphylaxis (FIA) has increased along with food allergies. In the United Kingdom, hospital admissions due to FIA doubled from 1998 to 2012.^7^ In Australia, the number of hospitalizations for anaphylactic shock among patients under the age of 5 years increased from 4.1 to 19.7 per 100,000 people between 1993–1994 and 2004–2005, for which the main cause was noted to be food.^8^

FIA can result in sudden death of healthy children and can have a significant social impact. In Japan, a fatal incident due to a milk allergy was reported following consumption of a school meal in 2012. Subsequently, the Japanese government enacted the Basic Law for Allergic Diseases and clarified the responsibilities of the government, local governments, citizens, healthcare workers, and school establishments. The law also promotes epidemiological, basic, and clinical research into allergic diseases.

Past epidemiological studies have shown that foods that cause serious symptoms differ among different regions. In the United States, a cross-sectional study of 3,339 children who had food allergies showed that tree nuts and peanuts are the most common causative food of severe allergic reactions.^6^ In Japan, eggs followed by milk and wheat are the most common causative foods across all ages.^3^ Among those under 20 years old, hen’s eggs, fish roe, fruit, and crustaceans are the most common causative food of new-onset food allergies for those aged 0–1, 2–3, 4–6, and 7–19 years old, respectively. The first- and second-most common causative foods of accidental ingestion are hen’s eggs and cow’s milk, respectively, among those aged 0–19 years old. A Japanese prospective study conducted between 2000 and 2001 reported that the most common causative foods of anaphylactic shock among 395 patients were hen’s egg, cow’s milk, wheat, buckwheat, and peanuts, in that order.^9^ However, there is yet to be a large-scale epidemiological survey on foods that cause anaphylactic shock in Japan.

Therefore, we investigated the causes and characteristics of patients hospitalized due to FIA using the Diagnosis Procedure Combination (DPC) database, a standardized electronic claims system adopted by hospitals throughout Japan.

## METHODS

### Study design

We performed a cross-sectional study using data from the Japanese DPC database from April 2014 to March 2017. All data in the DPC database were gathered by the DPC research group, which receives funding from the Japanese Ministry of Health, Labour and Welfare. A total of 1,478 hospitals completed a survey conducted by the DPC research group during the study period and allowed their DPC data to be used for research purposes. The DPC database harbors inpatient data and comprehensive procedures for the Japanese national health insurance system.^10^ This study was conducted with approval from the ethics committee of medical care and research of the University of Occupational and Environmental Health, Japan (approved number R1-067).

### Inclusion and exclusion criteria

We selected patients whose principal diagnosis was anaphylactic shock due to an adverse food reaction (ICD-10 code T78.0; *n* = 10,049), including food-dependent exercise-induced anaphylaxis. The diagnosis was determined by the physician when the patient was discharged from hospital. To homogenize patients’ background characteristics, patients who had scheduled hospitalizations were excluded because they may have been hospitalized for an oral food challenge rather than for treatment of the acute phase of anaphylactic shock (*n* = 970).

### Types of causative food

We analyzed incidences in which the following seven foods were the cause of anaphylactic shock because labeling for these foods is required by the Food Labeling Act in Japan: eggs, wheat, milk, peanuts, buckwheat, shrimp, and crab. The causative food was identified based on the disease name written in Japanese in combination with the ICD-10 code. A food was considered a causative food only when the principal diagnosis or comorbidity was anaphylactic shock due to adverse food reaction (ICD-10: T78.0) or other adverse food reactions not classified elsewhere (ICD-10: T78.1) and the causal food was clearly indicated in the diagnosis at the time of discharge.

### Other variables and outcomes

We examined sex and age because the incidence of food-induced anaphylactic shock differs among categories within these variables.^1^^,^^3^^,^^6^^–^^8^^,^^11^^–^^14^ IgE testing conducted during hospitalization was examined because it is widely used to confirm a diagnosis of FIA.^14^ We also examined tests on breathing and circulation, which are performed on patients with severe symptoms. Use of epinephrine injections, oxygen, and steroids on the first day were examined because these are often used for severe symptoms.^1^^,^^11^^,^^15^ Prescription for epinephrine self-injection on the day of discharge was used as a variable because this occurs when symptoms are severe.^16^ To determine whether there was a repetition of adverse events, we examined the occurrence of readmission due to another FIA episodes regardless of the causative foods. In the case of rehospitalization, we calculated the period between hospitalizations.

### Statistical analysis

We conducted a descriptive epidemiological study by age group. We also tabulated the findings according to causative foods. All statistical analyzes were performed using Stata version 15.1 (StataCorp, College Station, TX, USA).

## RESULTS

Table 1 shows the characteristics of all patients. The most common causative food among all cases was eggs (*n* = 608, 6.7%), followed by wheat (*n* = 436, 4.8%), milk (*n* = 416, 4.6%), peanuts (*n* = 287, 3.2%), and buckwheat (*n* = 185, 2.0%). The causative food for 79% of patients was not listed. We did not separate shrimp and crab into different groups in Table 1 because less than 10 patients were hospitalized for anaphylactic shock due to these foods during the study period. We also did not describe a number of fatal cases because less than 10 patients died in the study period. Table 2 shows the patients’ characteristics by age group. We divided age into the following groups: <1, 1, 2–3, 4–6, 7–19, and ≥20 years old, with reference to Japanese guidelines.^3^ Groups younger than the 20-year-old group tended to be comprised of fewer females. Over 30% of the causative foods examined were identified among patients 6 years old or younger. The most common causative food in each age group was eggs among 0–3-year-olds, milk among 4–6-year-olds, peanuts among 7–19-year-olds, and wheat among those 20 years and older. IgE testing was most frequently performed among those under 1 year old, followed by those aged 7–19 years old. The proportion of patients using epinephrine at the time of admission was 38–50%. The proportion who used epinephrine two or more times or oxygen tended to increase with age. Epinephrine self-injection was prescribed to those aged 2–3 years and older, and the rate of prescription increased with age among those under 20 years. The length of hospital stay was approximately 2 days for all age groups. The readmission rate was 2.4%, 5%, 6%, and 3.1% for those aged <1, 1–3, 4–19, and over 20 years old. The period until rehospitalization varied widely.

**Table 1.** Characteristics of patients, type of causative food and treatment

		Number or mean	% or SD
Sex, female		3,930	43%
Causative food		
	Seven items required by law^a^	1,862	21%
	Other items	52	0.6%
	Not specified	7,165	79%
Type of causative food			
	Hen’s egg	608	6.7%
	Wheat	436	4.8%
	Cow’s milk	416	4.6%
	Peanuts	287	3.2%
	Buckwheat	185	2.0%
Clinical test			
	Blood gas analysis, yes	4,160	46%
	Percutaneous arterial oxygen saturation, yes	4,040	44%
	Respiratory and heart rate monitoring, yes	4,959	55%
	Non-specific IgE, yes	2,776	31%
	Specific IgE, yes	2,898	32%
Treatment on the first day			
	Use of epinephrine injections, yes	4,191	46%
	Use of epinephrine injections 2 times or more, yes	243	2.7%
	Use of oxygen, yes	2,775	31%
	Use of steroids, yes	6,023	66%
Prescription for epinephrine self-injection on the day of discharge, yes		427	4.7%
Length of stay, days, mean (SD)		2.1	(1.5)
Readmission (excluding scheduled hospitalization), yes		400	4.4%
Period until rehospitalization, days, mean (SD)		261	(264)

IgE, immunoglobulin E; SD, standard deviation.

^a^Seven items required by law: hen’s egg, wheat, cow’s milk, peanuts, buckwheat, shrimp and crab.

**Table 2.** Characteristics of patients, type of causative food and treatment by age group

		<1 years old		1 years old		2–3 years old		4–6 years old		7–19 years old		≥20 years old	
		*n* = 838		*n* = 618		*n* = 1,050		*n* = 845		*n* = 2,140		*n* = 3,587	
					
		Number or mean	% or SD	Number or mean	% or SD	Number or mean	% or SD	Number or mean	% or SD	Number or mean	% or SD	Number or mean	% or SD
Sex, female		346	41	217	35	358	34	266	31	875	41	1,869	52
Causative food													
	Seven items required by law^a^	367	44	203	33	351	33	268	32	347	16	326	9.1
Type of causative food													
	Hen’s egg	228	27	109	18	108	10	68	8.0	74	3.5	21	0.6
	Wheat	56	6.7	39	6.3	62	5.9	49	5.8	59	2.8	171	4.8
	Cow’s milk	92	11	59	9.5	105	10	77	9.1	>70	>3	<10	<1
	Peanuts	0		11	1.8	66	6.3	73	8.6	96	4.5	41	1.1
	Buckwheat	<10		<10		30	2.9	14	1.7	50	2.3	89	2.5
Clinical test													
	Blood gas analysis, yes	451	54	341	55	561	53	410	49	978	46	1,419	40
	Percutaneous arterial oxygen saturation, yes	317	38	240	39	422	40	353	42	951	44	1,757	49
	Respiratory and heart rate monitoring, yes	353	42	232	38	442	42	361	43	1,146	54	2,425	68
	Non-specific IgE, yes	505	60	251	41	333	32	235	28	748	35	704	20
	Specific IgE, yes	519	62	239	39	336	32	209	25	744	35	851	24
Treatment on the first day													
	Use of epinephrine injections, yes	318	38	261	42	508	48	350	41	947	44	1,808	50
	Use of epinephrine injections 2 times or more, yes	11	1.3	12	1.9	20	1.9	21	2.5	52	2.4	127	3.5
	Use of oxygen, yes	105	13	103	17	230	22	202	24	630	29	1,505	42
	Use of steroids, yes	462	55	378	61	606	58	492	58	1,383	65	2,702	75
Prescription for epinephrine self-injection on the day of discharge, yes		<10		<10		30	2.9	55	6.5	160	7.5	176	4.9
Length of stay, mean (SD)		2.2	(0.7)	2.1	(0.6)	2.1	(1.2)	2.1	(0.5)	2.1	(0.8)	2.2	(2.3)
Readmission (excluding scheduled hospitalization), yes		20	2.4	32	5.2	50	4.8	52	6.2	136	6.4	110	3.1
Period until rehospitalization, days		170	153	314	254	323	301	264	260	266	283	227	238

IgE, immunoglobulin E; SD, standard deviation.

^a^Seven items required by law: hen’s egg, wheat, cow’s milk, peanuts, buckwheat, shrimp and crab.

The results by causative food are shown in Table 3. Regardless of the causative food, women comprised around 40% and length of stay was about 2 days. Epinephrine was used at admission among about 40%, 50%, and over 60% of cases in which the causative food was eggs; wheat, milk, and peanuts; and buckwheat, respectively. About 8% of cases among whom the causative food was buckwheat used epinephrine two or more times. Cases among whom the causative food was eggs had the lowest rates of epinephrine, oxygen, and steroid use. The proportion of cases with a prescription for epinephrine self-injection at discharge was highest among those in which the causative food was wheat, followed by peanuts, buckwheat, milk, and eggs. The readmission rate was highest in cases in which the causative food was wheat, followed milk and eggs.

**Table 3.** Characteristics of patients and treatment by type of causative food

		Hen’s egg		Wheat		Cow’s milk		Peanuts		Buckwheat	
		*n* = 608		*n* = 436		*n* = 416		*n* = 287		*n* = 185	
				
		Number or mean	% or SD	Number or mean	% or SD	Number or mean	% or SD	Number or mean	% or SD	Number or mean	% or SD
Sex, female		241	40	162	37	153	37	112	40	75	41
Clinical test											
	Blood gas analysis, yes	275	45	219	50	217	52	137	48	76	41
	Percutaneous arterial oxygen saturation, yes	231	38	219	50	165	40	111	39	81	44
	Respiratory and heart rate monitoring, yes	254	42	248	57	193	46	138	48	106	57
	Non-specific IgE, yes	251	41	110	25	125	30	90	31	52	28
	Specific IgE, yes	251	41	112	26	116	28	84	29	55	30
Treatment on the first day											
	Use of epinephrine injections, yes	247	41	232	53	202	49	141	49	115	62
	Use of epinephrine injections 2 times or more, yes	<10	<1	17	3.9	10	2.4	<10	<2	15	8.1
	Use of oxygen, yes	110	18	169	39	112	27	69	24	78	42
	Use of steroids, yes	330	54	129	67	261	63	184	64	135	73
Prescription for epinephrine self-injection on the day of discharge, yes		13	2.1	31	7.1	21	5.1	18	6.3	10	5.4
Length of stay, mean (SD)		2.1	0.5	2.1	0.6	2.2	1.8	2.1	0.6	2.1	0.8
Readmission (excluding scheduled hospitalization), yes		19	3.1	31	7.1	17	4.1	<10	<2	<10	<2
Period until rehospitalization, days		225	217	244	231	208	189	N/A		N/A	

IgE, immunoglobulin E; N/A, not available; SD, standard deviation.

## DISCUSSION

This study used the Japanese DPC database to investigate the patient characteristics, causative food, and treatment for patients hospitalized due to FIA. We found that the proportion of cases in which the causative food was eggs, wheat, and milk decreased with increasing age. Cases due to peanuts peaked in those aged 4–6 years before declining. Buckwheat was the causative food in over 2% cases among 2–3-year-olds and remained at this rate thereafter.

Our study has three strengths. First, we analyzed a large sample from the Japanese DPC database, which includes a cumulative total of 33 million hospital admissions for all conditions across 4 years. A systematic review showed that there are 1 to 77 per 100,000 person-years of FIA.^17^ Large databases are useful for examining the epidemiology of rare diseases. A descriptive study conducted for 12 years since the 1990s with a sample size of about 6,000 is the largest study to date.^8^ The largest sample size in a published report since the 2010s is about 2,000 in the United States and about 3,000 in Europe.^5^^,^^6^ In contrast, our study enrolled about 9,000 severe cases from a single country across 4 years. Second, we used anonymized data that can be linked on an individual basis and evaluated incidence of readmission. All large-scale studies conducted in Australia, the United States, and Europe have been cross-sectional studies.^5^^,^^6^^,^^8^ Previous cohort studies on food allergies conducted in Japan involved immediate reactions other than anaphylaxis, and a sample size of about 5,000.^18^ In the DPC database, each patient is given the same anonymized ID when they revisit the same hospital, enabling rehospitalization to be detected on an individual basis. Third, the DPC database provided detailed medical practice information. A study conducted in the United States based solely on participants’ report highlighted recall bias as a limitation.^6^^,^^19^ The database we used is based on insurance claims and provides information about procedures and medications. No study to date has determined the status of IgE testing by age group or epinephrine, oxygen, or steroid use by causative food.

Our results differed from those of previous studies that examined the prevalence of immediate allergies in Japan. According to Japanese guidelines, the most common causative food is eggs, which was consistent with our results for anaphylactic shock.^3^ A cohort study conducted in Japan also showed that hen’s eggs were the most common causative food of food allergies among children aged 1 and 3 years old.^20^ In contrast, the prevalence of immediate wheat, milk, peanut, and buckwheat allergies is 30%, 55%, 13%, and 5.6% of that for eggs in the guidelines, compared to 72%, 68%, 47%, and 30% of that for eggs for FIA in our study. This result suggests that the severity of symptoms may vary depending on the causative food. In analysis by age, peanuts, like milk, were the most common causative food in the group comprising 4–6-year-olds, despite the fact that hen’s eggs, wheat, and cow’s milk are thought to be the three major causative foods in Japan. Peanut allergies, which are common in the United States, are also increasing in Japan.^21^ These results suggest that it is becoming increasingly important to check for peanuts in foods eaten by 4–6-year-olds, who are too young to choose the most appropriate foods for themselves. We also found that peanuts were the most common causative food among patients aged 7–19 years. A systematic review revealed that there is little evidence of food allergy prevention in older children or adults.^22^ Another qualitative study indicated the need to involve a wide range of people outside of family in the management of adolescent peanut allergies.^23^ The most recent school-lunch-related deaths in Japan were among upper elementary school children. Our results show that IgE testing is more frequently conducted in children aged 7–19 years old, after those aged under 1 year old. IgE testing is used to confirm a diagnosis of FIA, and the results suggest that the patient underwent anaphylactic shock in response to a food to which he or she has never had an allergic reaction. Our results suggest the importance of social strategies for preventing FIA in older children and young adults.

There were several limitations in this study. First, we used discharge diagnosis to identify causative foods. Previous studies have used the results of oral food challenge tests, skin prick tests, or other measures of food-specific IgE.^5^^,^^12^^,^^21^ In our study, laboratory test results were not available. In addition, the percentage of foods that were actually identified as being causative among the listed potential causative foods decreased with increasing age. Second, we may have underestimated the readmission rate because, while we could identify cases of readmission to the same hospital due to assignment of the same anonymized ID in the DPC database, it was impossible to capture rehospitalization when patients visited a different hospital. However, this problem can be solved by conducting a study using the National Database of Health Insurance Claims and Specific Health Checkups of Japan, the Japanese government’s repository for all health insurance claims data. Third, our study did not include patients who were judged not to require hospitalization after an emergency department visit. Therefore, we were unable to calculate population-based prevalence in the same way as epidemiological studies conducted in the United Kingdom that included inpatients.^7^ However, identifying the proportion of cases with relatively severe symptoms that require hospitalization due to certain foods is important for preventing death from FIA. Fourth, we were unable to examine any known factors that affect food allergy or FIA that were not indicated in the DPC data. A previous cohort study showed that parents’ history of allergies affects their children’s allergies.^24^ Further, a cohort study conducted in Japan has revealed the epidemiology of parental history of allergies.^25^ Future studies should use these data in an FIA study.

In summary, we identified the causative foods, details of treatment, and readmission rate of patients hospitalized for anaphylactic shock using data from the DPC database. While the breakdown of causative foods was similar to that reported by previous studies, a novel finding was that peanuts were a common causative food of FIA in children and teenagers in Japan, as they are in the West. These results suggest the importance of taking measures to prevent peanut allergies in preschool or school as well as FIA due to the three major causative foods. In the future, government efforts are needed to improve the reporting rate of causative foods for more accurate statistics.
